# PBX3-HMGCR Axis Promotes Hepatocellular Carcinoma Progression Through Enhancing De Novo Cholesterol Biosynthesis

**DOI:** 10.3390/ijms26115210

**Published:** 2025-05-29

**Authors:** Xia Zhang, Li Qiu, Lei Zhang, Wenfang Li, Debing Xiang, Jian Wang, Shourong Wu, Vivi Kasim

**Affiliations:** 1 Key Laboratory of Biorheological Science and Technology, Ministry of Education, College of Bioengineering, Department of Oncology, Chongqing University Jiangjin Hospital, Chongqing University, Chongqing 400044, China; zx2556915346@163.com (X.Z.); qiuli1230107@163.com (L.Q.); zhangleigloria@163.com (L.Z.); wenfang520@126.com (W.L.); xdb86@cqu.edu.cn (D.X.); wj929@cqu.edu.cn (J.W.); 2 The 111 Project Laboratory of Biomechanics and Tissue Repair, College of Bioengineering, Chongqing University, Chongqing 400044, China

**Keywords:** tumor metabolic reprogramming, lipid metabolism, cholesterol biosynthesis, PBX3, HMGCR

## Abstract

Tumor cells alter lipid metabolic pathways to meet their demands for energy and membrane biosynthesis. Despite its crucial role in tumor cell growth, survival, and metastasis, the mechanisms underlying tumor cell lipid metabolic reprogramming remain poorly understood. Pre-B-cell leukemia transcription factor 3 (PBX3), a member of the PBX family, could promote tumorigenesis; however, whether it is involved in tumor lipid metabolic reprogramming remains unknown. Herein, we found that PBX3 significantly promotes tumor growth by enhancing lipid accumulation in HCC cells. By assessing the effect of PBX3 on the expression levels of lipid metabolism-related genes, we found that PBX3 could positively regulate the expression of 3-hydroxy-3-methylglutaryl CoA reductase (HMGCR), a rate-limiting enzyme in the cholesterol biosynthesis pathway. Mechanistically, we revealed that PBX3 could directly bind to the −167/−151 region of *HMGCR* promoter, thereby increasing its transcriptional activity and, subsequently, its expression level. This leads to the increase of HCC cell cholesterol biosynthesis and, eventually, to the increase of the in vivo tumorigenic potential. Collectively, our research revealed an unprecedented regulatory mechanism of cholesterol metabolism in HCC cells through PBX3 positive regulation on HMGCR expression levels. These findings provide novel insights into tumor metabolic reprogramming and uncover a previously unknown physiological function for PBX3. Moreover, these results suggest the potential of targeting *PBX3* as an anti-tumor therapeutic strategy.

## 1. Introduction

Tumor can be regarded as a metabolic disorder characterized by dysregulated metabolic processes, which enable tumor cells to proliferate rapidly and survive in a harsh environment [1]. Metabolic reprogramming is one of the basic hallmarks of tumor cells essential for tumorigenesis and tumor progression [2]. Tumor cells alter their metabolism to provide not only energy but also building blocks to support their rapid growth. Furthermore, metabolic reprogramming also provides tumor cells with cellular reductants necessary for adaptation to tumor microenvironment [3]. Lipid is one of the fundamental cell components crucial for tumorigenesis. Lipid is not only a crucial energy source, but [4] also performs critical functions in signal transduction as well as metabolism [5,6]. Furthermore, lipid is a crucial component of cell membrane [7]. In normal cells, lipid homeostasis is regulated by the balance of lipid synthesis, which includes the synthesis of fatty acids triglycerides, phospholipids, as well as cholesterol, and fatty acid oxidation, especially fatty acid β-oxidation [8,9,10]. This balance is disrupted in various tumor cells, including breast cancer, colorectal cancer, hepatocellular carcinoma (HCC), pancreatic cancer, and non-small cell lung cancer [11,12]. Tumor cells alter their lipid metabolism by upregulating lipogenesis and downregulating lipid catabolism, thereby promoting lipid accumulation [13]. The increase in lipid accumulation in turn provides tumor cells with energy resources, membrane components, and signaling molecules, which eventually support their rapid proliferation [14]. However, despite their importance, the molecular mechanisms regulating tumor cell lipid metabolism have not been totally understood.

Previous studies revealed that leukemia transcription factor 3 (PBX3) could promote tumor cell growth, survival, migration, and invasion potential [15,16]. Aberrant upregulation of PBX3 has been observed in various cancers, including in acute myeloid leukemia [17] and in solid tumors, such as breast cancer [18], lung cancer [19], HCC [20], and colorectal cancer [21]. Concomitantly, PBX3 upregulation in clinical tumor patients is often associated with poor prognosis [22]. PBX3 is a homeodomain-containing transcription factor that belongs to the PBX family, a family of proteins with three α-helical rings extending over the homologous domain [18]. PBX3 is essential for normal embryonic development, affecting the formation of critical organs and systems such as the skeleton, heart, and nervous system [23]. Moreover, PBX3 could promote the expression of angiogenic factors, such as vascular endothelial growth factor and platelet-derived growth factor, thereby facilitating tumor angiogenesis [24]. Despite its important roles as an oncogene, the role of PBX3 in regulating tumor cell lipid metabolic reprogramming remains unknown.

In this study, to elucidate the role of PBX3 in regulating tumor cell lipid metabolic reprogramming, we investigated the effects of manipulating PBX3 expression on genes related with lipid metabolism and identified HMGCR as a novel target of PBX3. Through in vitro and in vivo experiments, we revealed that PBX3 regulation on *HMGCR* transcription promotes HCC cells cholesterol biosynthesis and, eventually, tumorigenesis. This study not only unravels a novel regulatory mechanism of HCC cells cholesterol metabolism but also provides new insights regarding the mechanism underlying the oncogenic function of PBX3. Furthermore, our findings also suggest the potential of targeting PBX3 for anti-tumor therapeutic strategy.

## 2. Results

### 2.1. PBX3 Alters HCC Cell Lipid Accumulation

**Figure 1:**
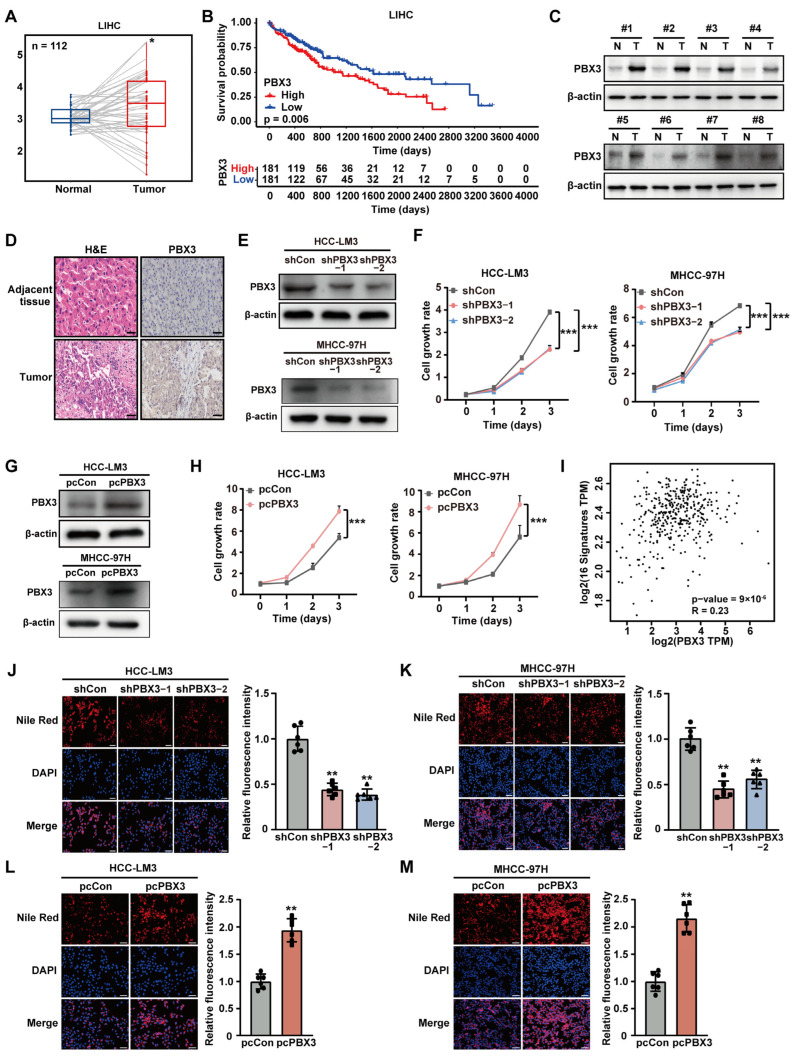
PBX3 promotes HCC cell lipid accumulation. (**A**) PBX3 expression level clinical sample datasets obtained from TCGA database. (**B**) Kaplan–Meier survival plots for clinical HCC patients obtained from TCGA database with either low (blue; *n* = 181) or high (red; *n* = 181) PBX3 expression. (**C**) PBX3 protein expression level in clinical human HCC and adjacent tissues samples, as determined using western blotting. (**D**) Expression levels of PBX3 in clinical human HCC and adjacent tissues samples, as examined by immunohistochemistry staining (scale bars: 50 μm). (**E**) PBX3 protein expression levels in HCC-LM3 and MHCC-97H cells transfected with two shRNA expression vectors targeting different sites of PBX3, as examined using western blotting. (**F**) Cell proliferation rates of *PBX3*-knocked down HCC-LM3 and MHCC-97H cells. (**G**) PBX3 protein expression levels in HCC-LM3 and MHCC-97H cells transfected with pcPBX3. (**H**) Cell proliferation rates of *PBX3*-overexpressing HCC-LM3 and MHCC-97H cells. (**I**) Correlation analysis of the expression levels of PBX3 and lipid metabolism-related genes in clinical HCC samples obtained from TCGA data set. (**J**,**K**) Lipid droplet accumulation in *PBX3*-knocked down HCC-LM3 (**J**) and MHCC-97H (**K**) cells, as determined using Nile Red staining. Representative images ((**left**); scale bars: 200 μm) and quantification results ((**right**); *n* = 6) are shown. (**L**,**M**) Lipid droplet accumulation in *PBX3*-overexpressing HCC-LM3 (**L**) and MHCC-97H (**M**) cells, as determined using Nile Red staining. Representative images ((**left**); scale bars: 200 μm) and quantification results ((**right**); *n* = 6) are shown. Cells transfected with shCon or pcCon were used as controls. β-actin was used as western blotting loading control. Quantification data are expressed as mean ± SD (*n* = 3; unless otherwise indicated). 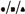 represent the data of each group. * *p* < 0.05; ** *p* < 0.01; *** *p* < 0.001.

To investigate the role of PBX3 in HCC cholesterol metabolism, we first analyzed its expression in clinical sample datasets obtained from the TCGA database. Based on the average expression of PBX3, the samples in the TCGA database were divided into high and low expression groups, and the expression of PBX3 in HCC and para-cancer was drawn. The results showed that PBX3 expression is upregulated in HCC (Figure 1A); furthermore, the PBX3 expression level significantly correlated with poor prognosis by calculating the Pearson correlation between PBX3 and HMGCR gene expression (Figure 1B). Western blotting and immunohistochemistry staining also confirmed that the expression level of PBX3 in clinical HCC lesions was significantly higher than in normal adjacent tissues (Figure 1C,D). Furthermore, knocking down *PBX3* (Figure S1 and Figure 1E) robustly suppressed the cell growth rate of HCC cell lines HCC-LM3 and MHCC-97H (Figure 1F); while overexpressing *PBX3* significantly enhanced it (Figure 1G,H). Together, these results demonstrate the oncogenic potential of PBX3 in HCC. 

Given the importance of lipid metabolism in highly proliferative tumor cells, we next assessed the possible involvement of PBX3 in tumor cell lipid metabolism. We first used the TCGA dataset to examine the correlation of PBX3 expression with 16 genes involved in lipid metabolism, i.e., fatty acid synthase (FASN), long chain acyl-coA synthetase 1 (ACSL1), long chain acyl-CoA synthetase 5 (ACSL5), and long chain acyl-coA synthetase 6 (ACSL6), which are key enzymes in fatty acid synthesis [25,26]; fatty acid-binding protein 3 (FABP3), and lipid phosphate phosphohydrolase 1 (LPIN1), which are related to lipid storage and release [27,28]; medium-chain acyl-CoA dehydrogenase (MCAD), short-chain acyl-CoA dehydrogenase (SCAD), long-chain acyl-CoA dehydrogenase (LCAD), short branched chain acetyl CoA dehydrogenase (SBCAD), peroxisomal trans-2-enoyl-CoA reductase (PECR), carnitine palmitoyl transferase 1A (CPT1A), and carnitine palmitoyl transferase 1B (CPT1B), which are involved in fatty acid oxidation [29]; 3-hydroxy-3-methylglutaryl-CoA reductase (HMGCR) and sterol regulatory element-binding protein (SREBP), which play vital roles in cholesterol biosynthesis [30]; and PPARγ, which could govern different processes involved in lipid metabolism [31]. The results showed a significant positive correlation between the expression of PBX3 with these genes, indicating that PBX3 might be correlated with tumor cell lipid metabolism (Figure 1I and Figure S2). To validate this possibility, we knocked down *PBX3* in HCC-LM3 and MHCC-97H cells and examined its effect on lipid accumulation. Knocking down *PBX3* suppressed the accumulation of lipid droplets in HCC-LM3 and MHCC-97H cells (Figure 1J,K), while overexpressing *PBX3* significantly increased it (Figure 1L,M). Together, these results indicate that PBX3 might promote tumor cell lipid metabolism.

### 2.2. PBX3 Regulates HCC Cell Cholesterol Metabolism by Promoting HMGCR Expression

**Figure 2:**
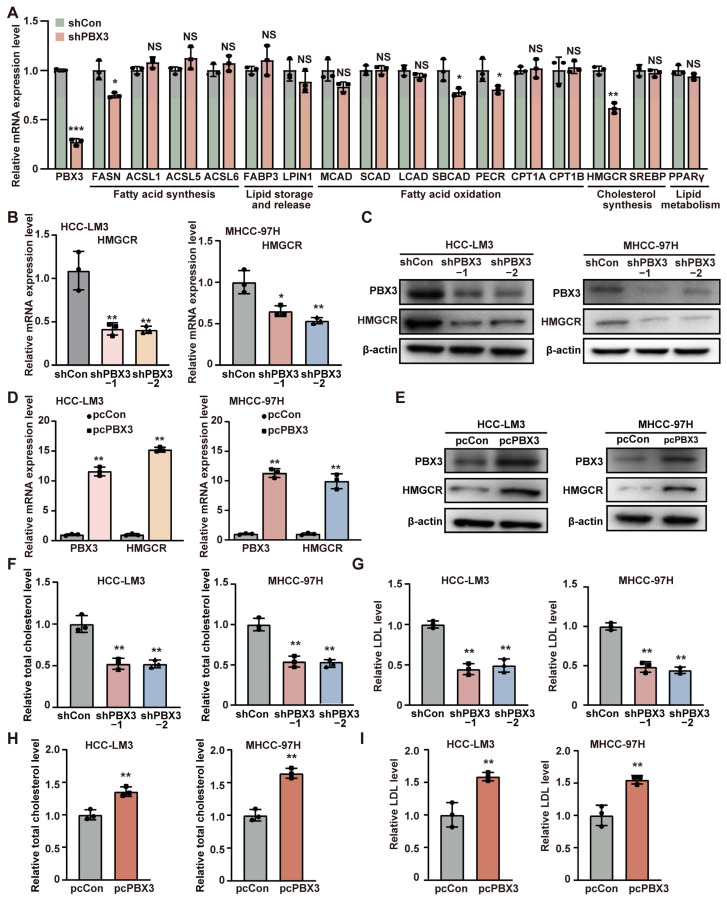
PBX3 promotes HCC cell cholesterol metabolism. (**A**) The mRNA expression levels of lipid metabolism-related genes in *PBX3*-knocked down HCC-LM3 cells, as determined using qRT–PCR. (**B**) HMGCR mRNA expression levels in *PBX3*-knocked down HCC-LM3 and MHCC-97H cells, as determined using qRT–PCR. (**C**) HMGCR protein expression levels in *PBX3*-knocked down HCC-LM3 and MHCC-97H cells, as determined using western blotting. (**D**) HMGCR mRNA expression levels in *PBX3*-overexpressing HCC-LM3 and MHCC-97H cells, as determined using qRT–PCR. (**E**) HMGCR protein expression levels in *PBX3*-overexpressing HCC-LM3 and MHCC-97H cells, as determined using western blotting. (**F**,**G**) Total cholesterol (**F**) and relative LDL (**G**) levels in *PBX3*-knocked down HCC-LM3 and MHCC-97H cells. (**H**,**I**) Relative total cholesterol (**H**) and LDL (**I**) levels in *PBX3*-overexpressing HCC-LM3 and MHCC-97H cells. Cells transfected with shCon or pcCon were used as controls. β-actin was used for qRT–PCR normalization and as western blotting loading control. Total protein was used for normalization of total cholesterol and LDL levels. Quantification data are expressed as mean ± SD (*n* = 3). 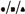 represent the data of each group. pcCon: pcEF9-Puro; * *p* < 0.05; ** *p* < 0.01; *** *p* < 0.001; NS: not significant.

To elucidate the molecular mechanism underlying PBX3 regulation on HCC lipid metabolism, we next investigated the effect of knocking down *PBX3* on the expression levels of the above mentioned lipid metabolism-related genes. As shown in Figure 2A, among the genes whose mRNA expression were affected by *PBX3* knockdown, HMGCR showed the most significant alteration. We next validated the effect of altering *PBX3* expression in HCC cells on HMGCR expression level. Knocking down *PBX3* robustly suppressed the mRNA and protein expression levels of HMGCR in both HCC-LM3 and MHCC-97H cells (Figure 2B,C), while overexpressing *PBX3* conspicuously increased it (Figure 2D,E). These results indicate that PBX3 is a positive regulator of HMGCR.

Given that HMGCR is the key enzyme of the mevalonate pathway, the first pathway of cholesterol biosynthesis, we next examine the effect of PBX3 alteration in cholesterol biosynthesis. Knocking down *PBX3* downregulated total cholesterol as well as LDL levels in HCC-LM3 cells and MHCC-97H cells (Figure 2F,G), while overexpressing *PBX3* resulted in the opposite (Figure 2H,I). These results suggest that PBX3 might be involved in cholesterol metabolism.

**Figure 3:**
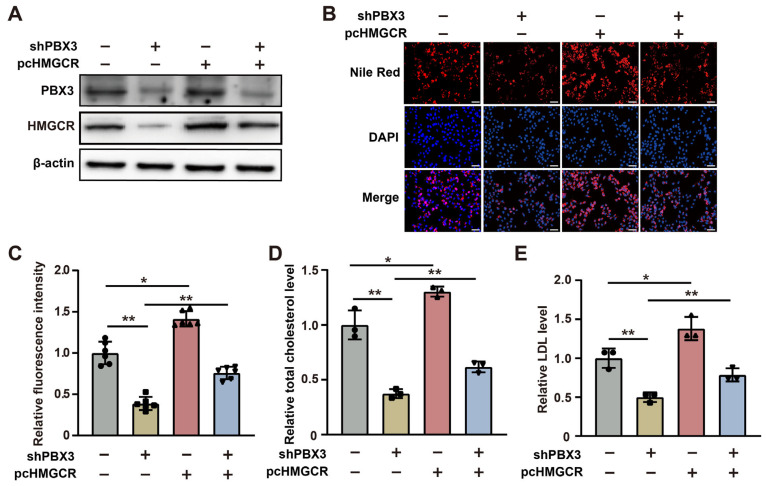
HMGCR is essential for PBX3-induced cholesterol biosynthesis. (**A**) PBX3 and HMGCR protein expression levels in *PBX3*-knocked down, *HMGCR*-overexpressing HCC-LM3 cells, as determined using western blotting. (**B**,**C**) Lipid droplet accumulation in *PBX3*-knocked down, *HMGCR*-overexpressing HCC-LM3 cells, as determined using Nile Red staining. Representative images ((**B**); scale bars: 200 μm) and quantification results ((**C**); *n* = 6) are shown. (**D**,**E**) Relative total cholesterol (**D**) and LDL (**E**) levels in *PBX3*-knocked down, *HMGCR*-overexpressing HCC-LM3 cells. Cells transfected with shCon and/or pcCon were used as controls. β-actin was used for western blotting loading control. Total protein was used for normalization of total cholesterol and LDL levels. Total protein was used for normalization of total cholesterol and LDL levels. Quantification data are expressed as mean ± SD (*n* = 3; unless otherwise indicated). 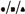 represent the data of each group. pcCon: pcEF9-Puro; * *p* < 0.05; ** *p* < 0.01.

Next, to elucidate whether PBX3 regulation of HMGCR is involved in its role as a positive regulator of cholesterol metabolism, we constructed an *HMGCR* overexpression vector (Figure S3) and overexpressed *HMGCR* in *PBX3*-knocked down HCC-LM3 cells (Figure 3A). Overexpression of *HMGCR* partially restored the lipid droplet accumulation suppressed by *PBX3* knockdown (Figure 3B,C). Similarly, *HMGCR* overexpression partially cancelled the effect of *PBX3* knockdown in suppressing total cholesterol and LDL levels (Figure 3D,E). Together, these results showed that PBX3 is a positive regulator of the HMGCR/mevalonate pathway, which in turn promotes cholesterol metabolism in HCC cells.

### 2.3. PBX3 Promotes HMGCR Transcriptional Activity

**Figure 4:**
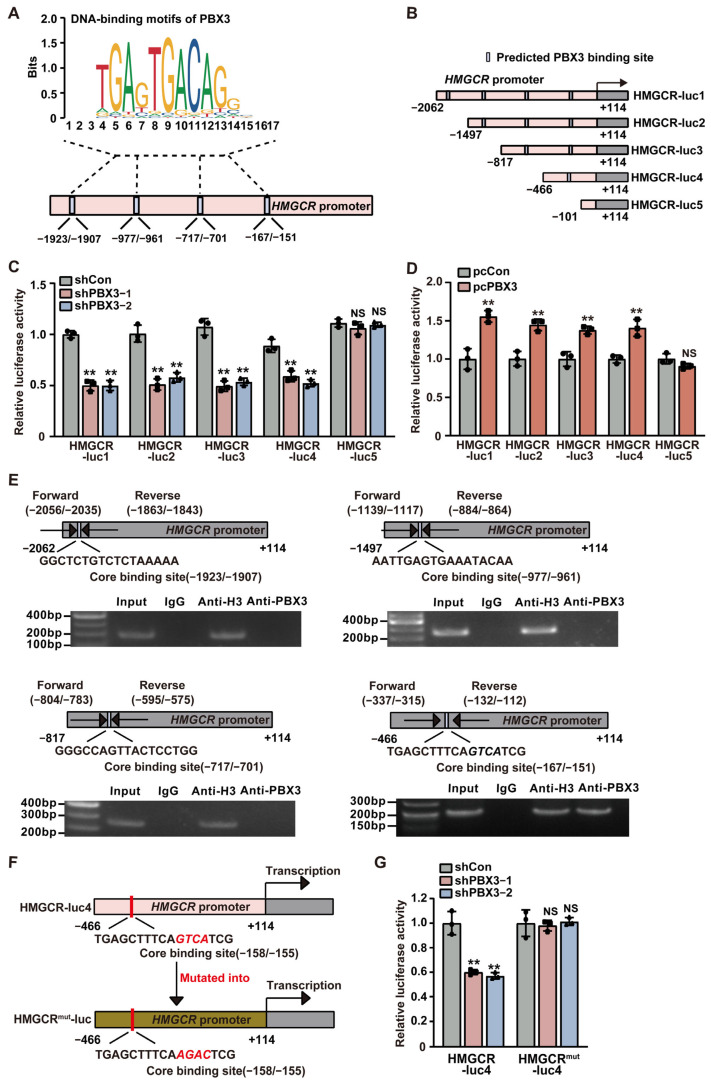
PBX3 directly binds to the *HMGCR* promoter and regulates its transcriptional activity. (**A**) Schematic diagram of the predicted PBX3 binding sites on the *HMGCR* promoter. (**B**) Schematic diagram of HMGCR reporter vectors (HMGCR-lucs). (**C**,**D**) Relative luciferase activities of HMGCR-luc1 to HMGCR-luc5 in *PBX3*-knocked down (**C**) and *PBX3*-overexpressing (**D**) HCC-LM3 cells. (**E**) Binding capacity of PBX3 to the predicted region on the *HMGCR* promoter in HCC-LM3 cells, as examined using ChIP assay with anti-PBX3 antibody followed by PCR. Predicted PBX3 binding site on the *HMGCR* promoter and the location of the primer set used for PCR are shown. Anti-histone H3 antibody was used as a positive control. (**F**,**G**) Relative luciferase activity of HMGCR-luc4^mut^ in *PBX3*-knocked down HCC-LM3 cells. Schematic diagram of HMGCR-luc4^mut^ (**F**) and luciferase activities (**G**) are shown. Cells transfected with shCon or pcCon were used as controls. Quantification data are expressed as mean ± SD (*n* = 3). 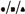 represent the data of each group. pcCon: pcEF9-Puro; ** *p* < 0.01; NS: not significant.

To further explore the molecular mechanism by which PBX3 regulates HMGCR, we next investigated the possibility of PBX3 binding to the *HMGCR* promoter. Prediction using a JASPAR database ((http://jaspar.genereg.net; accessed on 11 August 2023) revealed four possible PBX3 binding sequences in the −1923 to −1907, −977 to −961, −717 to −701, and −167 to −151 regions of the *HMGCR* promoter (Figure 4A). Accordingly, we constructed five luciferase reporter vectors carrying different regions of the *HMGCR* promoter: HMGCR-luc-1, which contained the −2062 to +114 region; HMGCR-luc-2, which contained the −1497 to +114 region; HMGCR-luc-3, which contained the −817 to +114 region; HMGCR-luc-4, which contained the −466 to +114 region; and HMGCR-luc-5, which contained the −101 to +114 region (Figure 4B). Luciferase assays revealed that knocking down *PBX3* significantly suppressed the activities of HMGCR-luc-1, HMGCR-luc-2, HMGCR-luc-3, and HMGCR-luc-4, but not that of HMGCR-luc-5 (Figure 4C). Concomitantly, *PBX3* overexpression significantly promoted HMGCR-luc-1, HMGCR-luc-2, HMGCR-luc-3 and HMGCR-luc-4 activities, while this did not significantly affect that of HMGCR-luc-5 (Figure 4D). Together, these results suggest that the −466 to −102 region of the *HMGCR* promoter is crucial for its transcriptional regulation by PBX3.

To determine whether PBX3 could bind directly to the putative PBX3-binding site in the *HMGCR* promoter region, we performed ChIP using an anti-PBX3 antibody and found that PBX3 specifically binds to the −337 to −112 region of the *HMGCR* promoter, but fails to bind to other predicted binding sites on the *HMGCR* promoter (Figure 4E). Subsequently, we constructed a mutant HMGCR luciferase reporter vector (HMGCR^mut^-luc) whereby the GTCA sequences of the predicted PBX3 core binding site located at the −158 to −155 region of the *HMGCR* promoter were mutated into AGAC (Figure 4F). The results showed that, whereas knocking down *PBX3* significantly suppressed the activity of HMGCR-luc4, it failed to alter the activity of HMGCR^mut^-luc (Figure 4G). Together, these results indicate that PBX3 could promote HMGCR expression by binding directly to the *HMGCR* promoter, thereby enhancing its transcriptional activity.

### 2.4. Cholesterol Is Critical for PBX3 Tumorigenic Potential

**Figure 5:**
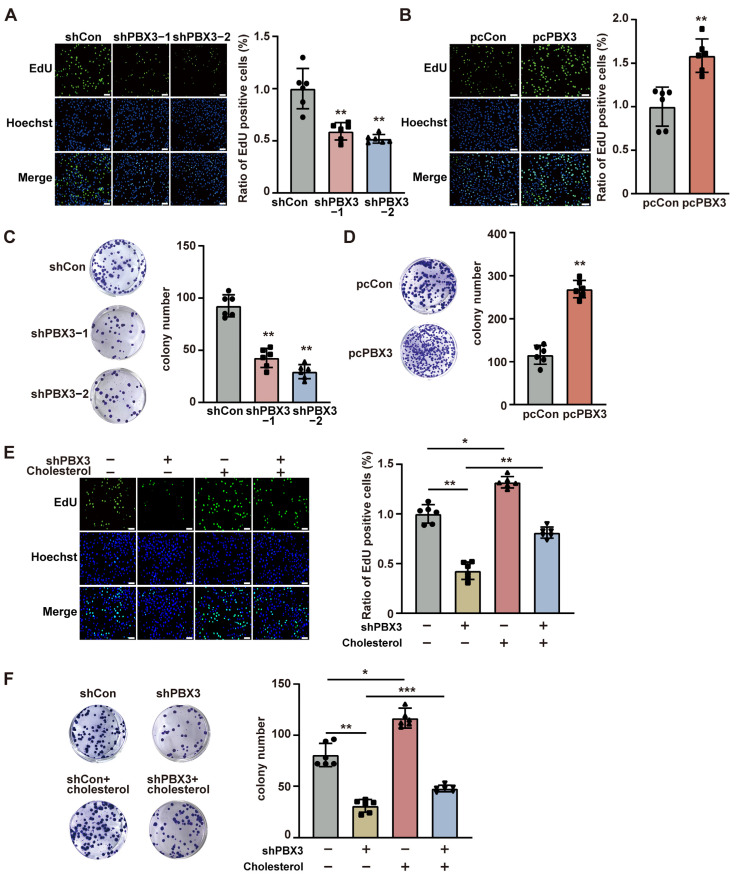
Cholesterol is critical for PBX3-induced HCC cell proliferation and colony formation. (**A**,**B**) Proliferation potential of *PBX3*-knocked down (**A**) and *PBX3*-overexpressing (**B**) HCC-LM3 cells, as determined using EdU-incorporation assay. Representative images ((**left**); scale bars: 100 μm) and quantification results ((**right**); *n* = 6) are shown. (**C**,**D**) Colony formation potential of *PBX3*-knocked down (**C**) and *PBX3*-overexpressing (**D**) HCC-LM3 cells. Representative images (**left**) and quantification results ((**right**); *n* = 6) are shown. (**E**) Proliferation potential of *PBX3*-knocked down HCC-LM3 cells treated with cholesterol (final concentration: 10 μg/mL), as determined using EdU-incorporation assay. Representative images ((**left**); scale bars: 100 μm) and quantification results ((**right**); *n* = 6) are shown. (**F**) Colony formation potential of *PBX3*-knocked down HCC-LM3 cells treated with cholesterol (final concentration: 10 μg/mL). Representative images (**left**) and quantification results ((**right**); *n* = 6) are shown. Cells transfected with shCon or pcCon were used as controls. Quantification data are expressed as mean ± SD. 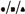 represent the data of each group. pcCon: pcEF9-Puro; * *p* < 0.05; ** *p* < 0.01; *** *p* < 0.001.

Given that cholesterol is crucial for tumorigenesis, we next examined the role of PBX3/HMGCR axis-mediated cholesterol biosynthesis on HCC tumorigenesis. Knocking down *PBX3* significantly suppressed the ratio of EdU-positive HCC-LM3 cells (Figure 5A), whereas its overexpression robustly increased it (Figure 5B). Similarly, a positive correlation was observed between the expression level of PXB3 and the EdU positivity rate of MHCC-97H cells (Figure S4A,B). Furthermore, the number of colonies formed by HCC-LM3 cells decreased significantly upon knocking down *PBX3* (Figure 5C) and increased partially following *PBX3* overexpression (Figure 5D). These results indicate that PBX3 is crucial for HCC cell proliferation and colony formation potentials.

Next, to understand the role of cholesterol in PBX3 regulation on HCC cell proliferation and colony formation potential, we supplemented exogenous cholesterol to the culture medium of *PBX3*-knocked down HCC cells. Cholesterol addition partially restored the ratio of EdU-positive cells in both *PBX3*-knocked down HCC-LM3 and MHCC-97H cells (Figure 5E and Figure S5). Concomitantly, cholesterol also partially cancelled the effect of knocking down *PBX3* on HCC-LM3 cells’ colony formation potential (Figure 5F). Together, these results showed that PBX3-regulated cholesterol biosynthesis is crucial for the tumorigenic potential of HCC cells.

### 2.5. PBX3/HMGCR Axis Is Crucial for HCC Tumorigenesis

**Figure 6:**
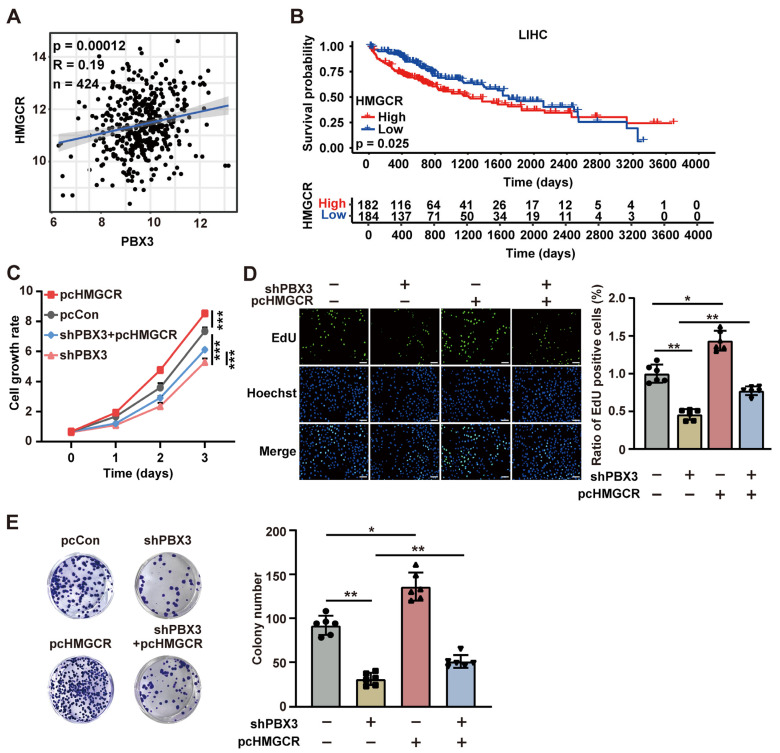
HMGCR is critical for PBX3-induced HCC cell proliferation potential. (**A**) Correlation analysis between PBX3 and HMGCR expression levels in HCC samples using TCGA data set. (**B**) Kaplan-Meier survival plots of clinical HCC patients with either low (blue; *n* = 184) or high (red; *n* = 182) PBX3 expression. (**C**) Cell growth rate of *PBX3*-knocked down, *HMGCR*-overexpressing HCC-LM3 cells. (**D**) Proliferation potential of *PPBX3*-knocked down, *HMGCR*-overexpressing HCC-LM3 cells, as determined using EdU-incorporation assay. Representative images ((**left**); scale bars: 100 μm) and quantification results ((**right**); *n* = 6) are shown. (**E**) Colony formation potentials of *PPBX3*-knocked down, *HMGCR*-overexpressing HCC-LM3 cells, Representative images (**left**) and quantification results ((**right**); *n* = 6) are shown. Cells transfected with shCon and/or pcCon were used as controls. Quantification data are expressed as mean ± SD (*n* = 3; unless otherwise indicated). 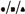 represent the data of each group. pcCon: pcEF9-Puro; * *p* < 0.05; ** *p* < 0.01; *** *p* < 0.001.

Subsequently, we assessed the role of HMGCR on the PBX3 regulation on HCC cells tumorigenic potential. Analysis using TCGA data set revealed that the expression levels of PBX3 and HMGCR showed positive correlation in clinical HCC lesions (Figure 6A). Furthermore, similar to PBX3, the HMGCR expression level also positively correlate with the poor prognosis of clinical HCC patients (Figure 6B), suggesting the positive correlation between HMGCR expression and clinical HCC progression. Compared with merely knocking down PBX3, simultaneously knocking down PBX3 and overexpressing HMGCR in HCC-LM3 cells had lower cell growth rate. *HMGCR* partially restored the cell growth rate of *PBX3*-knocked down HCC-LM3 cells (Figure 6C). Moreover, overexpressing *HMGCR* partially restored the proliferative potential of *PBX3*-knocked down HCC-LM3 cells, as indicated by the increase of EdU-positive cells (Figure 6D), as well as their colony formation potentials (Figure 6E).

**Figure 7:**
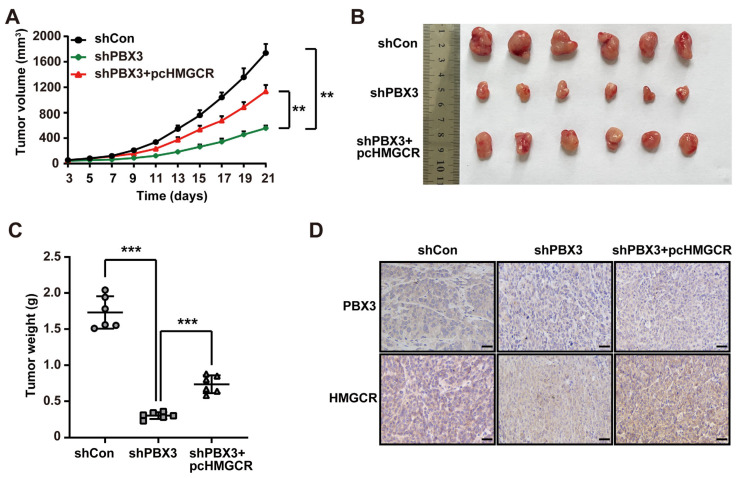

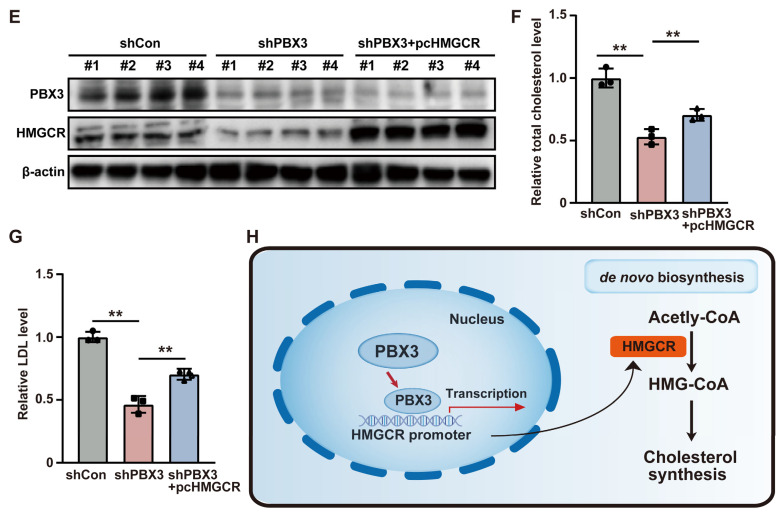
PBX3/HMGCR axis promotes HCC cells’ tumorigenic potential. (**A**–**C**) Tumorigenic potential of *PBX3*-knocked down, *HMGCR*-overexpressing HCC-LM3 stable cells, as examined in vivo by subcutaneous injection into BALB/c-nu/nu mice (*n* = 6). Volume of the tumors formed at indicated time-points (**A**), morphological images of the tumors generated (**B**), and tumor weight (**C**) at 21 days after injection are shown. (**D**) PBX3 and HMGCR expression levels in the xenograft tumors formed by *PBX3*-knocked down, *HMGCR*-overexpressing HCC-LM3 cells, as examined by immunohistochemistry staining using serial sections, (scale bars: 50 μm). (**E**) PBX3 and HMGCR protein expression levels in the xenograft tumors formed by *PBX3*-knocked down, *HMGCR*-overexpressing HCC-LM3 cells, as determined using western blotting. (**F**,**G**) Total cholesterol (**F**) and LDL (**G**) levels in the xenografted tumors formed by *PBX3*-knocked down, *HMGCR*-overexpressing HCC-LM3 cells. (**H**) Schematic diagram showing the molecular mechanism of PBX3/HMGCR regulation on tumor cells cholesterol synthesis. Cells transfected with shCon and/or pcCon were used as controls. β-actin was used for western blotting loading control. Total protein was used for normalization of total cholesterol and LDL levels. Quantification data are expressed as mean ± SD (*n* = 3). 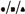 represent the data of each group. pcCon: pcEF9-Puro; ** *p* < 0.01; *** *p* < 0.001.

Furthermore, we analyzed the role of PBX3/HMGCR in regulating HCC tumorigenic potential in vivo by performing xenograft experiments using *PBX3*-knocked down, *HMGCR*-overexpressed HCC-LM3 stable cell line. As shown in Figure 7A, knocking down *PBX3* significantly slowed down the growth of the xenografted tumors formed by HCC-LM3 cells, whereas *HMGCR* overexpression partially reduced this suppressive effect, thus partially restoring the tumor growth rate as well as the size and weight of the tumors (Figure 7B,C). Immunohistochemistry and western blotting results showed that HMGCR expression was suppressed in the xenografted tumors formed by *PBX3*-knocked down HCC-LM3 cells (Figure 7D,E). Concomitantly, the total cholesterol and LDL levels were downregulated in these tumor lesions (Figure 7F,G). Meanwhile, overexpression of *HMGCR* partially reduced these effects. These findings indicate that PBX3/HMGCR promotes HCC cells’ tumorigenic potential in vivo.

In summary, our study indicates that PBX3 stimulates cholesterol biosynthesis in HCC cells by directly activating the transcription of *HMGCR*, the rate-limiting enzyme of mevalonate pathway. Furthermore, PBX3-mediated cholesterol biosynthesis activation is crucial for its oncogenic function, most plausibly by enhancing the tumorigenic potential of HCC cells (Figure 7H).

## 3. Discussion

Tumor cell metabolic reprogramming, including lipid metabolic reprogramming, is one of the most prominent characteristics of cancer that serves as a basis for tumor occurrence, tumor growth, metastasis, and drug resistance. Recent studies have shown that lipid metabolic reprogramming, including alteration in cholesterol metabolism, plays pivotal roles in tumorigenesis [32]. Cholesterol is a lipid molecule consisting of four carbon rings and 27 carbon atoms [30]. Cholesterol not only is a major component of the cell membrane but also serves as a fundamental precursor for the biosynthesis of vital physiological substances within the organism, such as bile acids, vitamin D, and corticosteroids. Cholesterol also plays a crucial role in supporting signal transduction [33], as it facilitates the aggregation of signaling molecules and receptors, thereby enhancing the precision and efficiency of the process. For instance, in the signal transduction pathway involving G protein-coupled receptors, cholesterol can significantly strengthen the interaction between the receptor and the G protein. This enhancement is vital for the effective transmission of downstream signals [34]. Furthermore, it could serve as a precursor for the synthesis of steroid hormones, bile acids, vitamin D, and lipoproteins [35]. Elevated cholesterol levels are often found in various tumors, including HCC, breast cancer, and prostate cancer [36], and are indispensable for tumor cell rapid proliferation, tumor progression, metastasis, as well as tumor drug resistance [37]. As a result, cholesterol metabolism has gained attention as a potential target for anti-tumor therapy [38]. HMGCR serves as a key enzyme that controls the rate of the cholesterol biosynthesis pathway, which plays a vital role in the synthesis of cholesterol [39]. SREBP and HMGCR closely cooperate in regulating the cholesterol biosynthesis pathway [40]. In the tumor microenvironment, activation of SREBP enhances cholesterol synthesis in tumor cells, influencing their metabolism. When cells’ demand for lipids and cholesterol increases, SREBP is activated and enters the nucleus, where it binds to the specific cis-acting elements in the HMGCR gene promoter, thereby promoting the transcription of HMGCR and cholesterol synthesis [41]. Our findings suggest that PBX3 could regulate tumor cell cholesterol biosynthesis by regulating the transcriptional activity of *HMGCR*, thereby unraveling a novel regulatory pathway of cholesterol metabolism in tumor cells. Notably, while further investigations are needed, PBX3 might also affect the expression of other lipid metabolism-related genes, such as FASN, SBCAD, and PECR, although less significant than its effect on HMGCR.

PBX3 plays a pivotal role in tumor cell proliferation, apoptosis, and migration by regulating various cellular processes [42]. PBX3 could promote tumor cell proliferation by activating the MAPK/ERK and PI3K/Akt signaling pathways [43], as well as by suppressing the transcription of tumor suppressor gene *p53* [44]. PBX3 could also promote the expression of Bcl-2, leading to the decrease in tumor cell apoptosis [45]. Furthermore, PBX3 could affect the expression of genes associated with cytoskeletal remodeling and extracellular matrix degradation, such as MMPs and vimentin, thereby promoting tumor cells’ metastatic potential [46]. Moreover, our previous research demonstrated that PBX3 could elevate G6PD expression, which in turn facilitates tumor cell glucose metabolic reprogramming through activating the pentose phosphate pathway [47]. However, the role of PBX3 in tumor cell lipid metabolism remains unclear. In this study, we revealed a novel function of PBX3 as a positive regulator of tumor cell cholesterol biosynthesis that promotes the transcriptional activity of *HMGCR*, and that PBX3/HMGCR-mediated cholesterol biosynthesis is crucial for its oncogenic activity. These results not only uncover a new regulatory pathway involved in tumor cell cholesterol metabolism but also provide insights into the molecular mechanisms driving the oncogenic activity of PBX3.

In summary, our findings reveal a new regulatory role of PBX3 in modulating HMGCR expression in HCC cells, establishing a connection between the oncogenic activity of PBX3 and tumor cell lipid metabolic reprogramming. This research offers new perspectives on the regulatory processes involved in cholesterol biosynthesis and enhances our understanding of the biological and pathological roles of PBX3. Furthermore, our findings demonstrate the potential of targeting PBX3 as an anti-tumor therapeutic strategy.

## 4. Materials and Methods

### 4.1. Vector Construction

Two shRNA expression vectors targeting different sites of *PBX3* were constructed as previously reported [44]. The target sequences are as follows: shPBX3-1: 5′-GGT CAA GGT TTA ATA TTG T-3′; shPBX3-2: 5′-GGG GAA ATG TGA ATA GGC A-3′. *PBX3* and *HMGCR* overexpression vector (pcPBX3 and pcHMGCR, respectively) were constructed as described previously [48]. The corresponding region was then amplified from the cDNA using Takara PrimeSTAR Max DNA Polymerase (Takara Bio, Kusatsu City, Japan), and the amplicon was then inserted into pcEF9-Puro vector [49]. For HMGCR luciferase reporter vectors, the −2062 to +114 (HMGCR-luc-1), −1497 to +114 (HMGCR-luc-2), −817 to +114 (HMGCR-luc-3), −466 to +114 (HMGCR-luc-4), and −101 to +114 (HMGCR-luc-5) regions of the *HMGCR* promoter were cloned into the *Nhe*I and *Stu*I sites of pGL4.13 vector (Promega, Madison, WI, USA). HMGCR-luciferase vector with mutated PBX3 binding site (HMGCR^mut^-luc) was constructed using a Site-directed Gene Mutagenesis Kit (Beyotime Biotechnology, Shanghai, China) based on the site-specific mutagenesis method.

### 4.2. Cell Lines and Cell Cultures

Wild-type human hepatocellular carcinoma cell lines, HCC-LM3 and MHCC-97H, were obtained from the Cell Bank of the Chinese Academy of Sciences (Shanghai, China) and cultured in McCoy’s 5A medium (Gibco, Life Technologies, Grand Island, NY, USA) supplemented with 10% FBS (Biological Industries, Beit Haemek, Israel) and 1% penicillin-streptomycin. All transfections were performed using Lipofectamine 2000 (Invitrogen Life Technologies, Carlsbad, CA, USA) in accordance with the manufacturer’s instructions. For gene knockdown and overexpression experiments, cells were seeded in 6-well plates and transfected with 2 µg of the indicated shRNA expression vector or overexpression vector. Twenty-four hours post-transfection, cells were subjected to puromycin selection (final concentration: 1 µg/mL) to eliminate untransfected cells. For rescue experiments, cells were seeded in 6-well plates and transfected with 1 μg shRNA expression vector and 1 µg overexpression vector before being subjected to puromycin selection. For establishment of *PBX3*-knocked down HCC-LM3 (HCC-LM3/shPBX3) and *PBX3*-knocked down, *HMGCR*-overexpressing HCC-LM3 (HCC-LM3/shPBX3/pcHMGCR) stable cells, cells were seeded in 10 cm culture plate and transfected with 12 μg of shCon or shPBX3 and 6 μg of pcCon or pcHMGCR vectors. Twenty-four hours later, puromycin selection (final concentration: 1.0 μg/mL) was performed to eliminate untransfected cells for 7 days.

### 4.3. Nile Red Staining

Cells were fixed using 4% paraformaldehyde and stained with 0.05 mg/mL Nile Red (Sigma-Aldrich, St. Louis, MO, USA) for 30 min. Nuclei were stained with DAPI. Images were taken using Olympus IX7I (Olympus, Tokyo, Japan). Quantitative analysis was performed using ImageJ 2.X (NIH, Bethesda, MD, USA). Fluorescence intensities were normalized by the corresponding cell number.

### 4.4. RNA Extraction and Quantitative Reverse Transcribed–PCR (qRT–PCR)

Total RNA was extracted using Trizol (Invitrogen Life Technologies) according to the manufacturer’s instructions. Total RNA sample (1 μg) was reverse transcribed into cDNA using PrimeScript Reagent Kit with gDNA Eraser (Takara Bio), then qRT–PCR was performed using SYBR Premix Ex Taq (Takara Bio). The sequences of the primers used for qRT–PCR are listed in Supplementary Table S1. β-actin was used to normalize sample amplification. The results are shown as relative to the expression level in the corresponding controls, which were assumed as 1.

### 4.5. Western Blotting

Cells were collected and lysed with RIPA lysis buffer supplemented with a protease inhibitor and phosphatase inhibitor cocktail (complete cocktail; Roche Applied Science, Mannheim, Germany). Equal amounts of samples (20 μg) were subjected to electrophoresis on sodium dodecyl sulfide polyacrylamide gels and transferred to polyvinylidene fluoride membrane with a pore size of 0.45 μm (Millipore, Billerica, MA, USA). PVDF membranes are blocked using 5% non-fat dry milk in Tris Buffered Saline Tween-20 (TBST) for 2 h. Membrane was then incubated with primary antibodies, which specifically recognize and bind to the target protein, and washed thoroughly with phosphate-buffered saline (PBS) to remove any unbound antibodies. Subsequently, the membrane was then incubated with secondary antibodies, which were conjugated to horseradish peroxidase (HRP). These secondary antibodies specifically bind to the primary antibodies, amplifying the signal for detection. The membrane was then washed again to remove any unbound secondary antibodies. Immunoblotting with anti-β-actin antibody was conducted to ensure equal protein loading. Signals were detected using SuperSignal West Femto Maximum Sensitivity Substrate detection system (Thermo Scientific, Waltham, MA, USA).

### 4.6. 5-Ethynyl-2′-deoxyuridine (EdU) Incorporation Assay and Colony Formation Assay

Cells were transfected with indicated vectors and selected using puromycin as indicated above. Cells were then re-seeded in a 24-well plate (1 × 10^5^ cells/well). EdU incorporation assay was performed using BeyoClick EdU Cell Proliferation Kit with Alexa Flour 488 (Beyotime Biotechnology) according to the manufacturer’s instructions. Images were taken with a fluorescence microscope (Olympus IX71, Tokyo, Japan). Quantification of EdU positive and Hoechst positive cells was performed using Image J, and the results are shown as the ratio of EdU positive cells to Hoechst positive cells.

### 4.7. Measurements of Total Cholesterol Level and Low-Density Lipoprotein (LDL) Levels

Total cholesterol and LDL levels were measured using the Total Cholesterol Assay Kit and Low-density Lipoprotein Assay Kit (Nanjing Jiancheng Bioengineering Institute, Nanjing, China), respectively. Briefly, cells were lysed with 3% Triton-X100 for 45 min, and supernatants were collected by centrifugation. The total cholesterol and LDL levels were then measured according to the manufacturer’s instruction. Total protein was used for normalization of total cholesterol and LDL levels.

### 4.8. Cell Growth Rate Assay

Cells were prepared as described above and re-seeded in 96-well plates at a density of 3 × 10^3^ cells/well. Cell numbers were measured at indicated time points using a colorimetric assay with 3-(4,5-dimethylthiazol-2-yl)-5-(3-carboxymethoxyphenyl)-2-(4-sulfophenyl)-2H-tetrazolium (Beyotime Biotechnology, China) in accordance with the manufacturer’s instructions.

### 4.9. Colony Formation Assay

Cells were prepared as described above and re-seeded into 6-well plates at a density of 300 cells/well. After being cultured for 15 days, cells were then fixed with 4% paraformaldehyde and stained with Crystal Violet Staining Solution (Beyotime Biotechnology). The numbers of the colonies formed were then counted. The investigator was blinded during the assessment.

### 4.10. Dual Luciferase Reporter Assay

Cells were co-transfected with indicated vectors, reporter vector bringing the firefly luciferase, and *Renilla* luciferase expression vector pRL-SV40 (Promega). After 24 h, luciferase activities were analyzed using Dual Luciferase Reporter Assay (Promega). The activities of the firefly luciferase reporters were normalized using those of *Renilla* luciferase.

### 4.11. Chromatin Immunoprecipitation (ChIP) Assay

ChIP assay was performed using ChIP-IT Express (Beyotime Biotechnology) according to the manufacturer’s instructions. Briefly, cells were cross-linked to 1% formaldehyde after 24 h of culture. The cells were then lysed, and the chromatin was sheared into small fragments of 200–400 bp by sonication. Chromatins were then immunoprecipitated using protein A + G Agarose/salmon sperm DNA and anti-PBX3 antibody, or normal mouse IgG, de-crosslinked for 4 h at 65 °C, and treated with 0.5 M EDTA, 1 M Tris pH 6.5 and 20 mg/mL proteinase K. Immunoprecipitated chromatin was then subjected to PCR using PrimeSTAR Max (Takara Bio). Primer sequences used for amplifying the *HMGCR* promoter region with the predicted PBX3 binding site were: 5′-AGG TGA CCG GAG CCA TTT ACA C-3′ (forward); and 5′-AAG CAG AAT CGG AGC GTG AG-3′ (reverse).

### 4.12. Animal Experiment

For in vivo tumor study, BALB/c-nu/nu mice (male; body weight: 18–22 g; 6 weeks old) were purchased from Chongqing University Cancer Hospital, (Chongqing, China). Animal study was approved by the Laboratory Animal Welfare and Ethics Committee of Chongqing University Cancer Hospital. All animal experiments conformed to the approved guidelines of the Animal Care and Use Committee of the Chongqing University Cancer Hospital. All efforts were made to minimize suffering. For xenograft experiments, BALB/c-nu/nu mice were randomly divided into three groups (*n* = 6), and each group was injected subcutaneously with 4 × 10^6^ of indicated stable cells. Tumor size (V) was evaluated by a caliper every 2 days with reference to the following equation: V = a × b^2^/2, where a and b are the major and minor axes of the tumor, respectively. The investigator was blinded to the group allocation and during the assessment.

### 4.13. Clinical Human HCC Specimens

Clinical human HCC specimens were obtained from HCC patients undergoing surgery at Chongqing University Cancer Hospital (Chongqing, China) and stored in the Biological Specimen Bank of Chongqing University Cancer Hospital. Patients did not receive chemotherapy, radiotherapy, or other adjuvant therapies prior to the surgery. The specimens were snap-frozen in liquid nitrogen. Prior patient’s written informed consents were obtained. The experiments were approved by the Laboratory Animal Welfare and Ethics Committee of Chongqing University Cancer Hospital and were conducted in accordance with the Declaration of Helsinki.

### 4.14. Immunohistochemistry and Hematoxylin and Eosin (H&E) Stainings

Fresh human HCC tissues, normal adjacent tissues, and xenografted tumors were fixed using 4% paraformaldehyde for overnight prior to being embedded in paraffin and sectioned at 4 μm thickness using a cryostat. Sections were then dewaxed using xylene and rehydrated before being incubated with primary antibodies for 1 h, followed by corresponding secondary antibodies conjugated with horse-radish peroxidase for 1 h. Visualization was performed using a DAB Kit (DAKO, Beijing, China) under microscope. Nuclei were then counterstained with hematoxylin (Beyotime Biotechnology), followed by dehydration and coverslip mounting. The antibodies used were listed in Supplementary Table S2. Images were taken using Pannoramic Midi (3DHistech, Budapest, Hungary). For H&E staining, paraffin sections were fixed with 10% formalin and washed with 60% propylene glycerol. Sections were then stained with Hematoxylin and Eosin (Sangon Bio, Shanghai, China) for 3 min followed by dehydration and coverslip mounting. Images were taken using Pannoramic Midi (3DHistech).

### 4.15. Statistical Analysis

All quantification results were presented as mean ± SD (*n* = 3, unless otherwise indicated). Statistical analysis was performed using two-tailed unpaired Student’s t-test conducted using GraphPad Prism 9.0. When more than two groups were compared, one-way ANOVA analyses were performed. A value of * *p* < 0.05 was considered statistically significant.

## Data Availability

The data that support the findings of this study are available from the corresponding authors upon reasonable request.

## Supplementary Materials

The following supporting information can be downloaded at: https://www.mdpi.com/article/10.3390/ijms26115210/s1.
