# Supplementary material for: PBX3-HMGCR Axis Promotes Hepatocellular Carcinoma Progression Through Enhancing De Novo Cholesterol Biosynthesis
Source: Int J Mol Sci. 2025 May 29;26(11):5210. doi: 10.3390/ijms26115210 (PMC12154334; doi:10.3390/ijms26115210)
Supplement: Supplementary file 1 [file ijms-26-05210-s001.zip › ijms-3520022-supplementary.pdf]

Supporting information for

PBX3-HMGCR axis promotes hepatocellular carcinoma progression through enhancing *de novo* cholesterol biosynthesis

Xia Zhang, Li Qiu, Lei Zhang, Wenfang Li, Debing Xiang, Jian Wang,

Shourong Wu*, Vivi Kasim*,

*E-mail: shourongwu@cqu.edu.cn (S.W.)
vivikasim@cqu.edu.cn (V.K.)

This PDF file includes:

Supplemental Figure S1: Efficacy of shRNA expression vectors targeting *PBX3*.

Supplemental Figure S2: Correlation of PBX3 and lipid metabolism-related genes in HCC.

Supplemental Figure S3: Efficacy of *HMGCR* overexpression vectors.

Supplemental Figure S4: PBX3 promotes HCC cells proliferation potential.

Supplemental Figure S5: Cholesterol is crucial for PBX3 regulation on HCC cells proliferation potential.

Supplemental Table S1: Primer pairs used for qRT-PCR.

Supplemental Table S2: Antibodies used for western blotting, ChIP assay and immunohistochemistry staining.

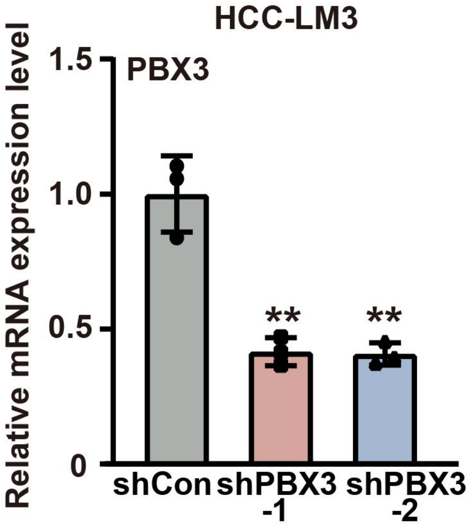

Supplemental Figure S1: Efficacy of shRNA expression vectors targeting *PBX3*.

PBX3 mRNA expression level in HCC-LM3 cells transfected with two shRNA expression vectors targeting different sites of *PBX3*, as analyzed using qRT-PCR. Cells transfected with shCon was used as controls. β-actin was used for qRT-PCR normalization. Quantification data are expressed as mean ± SD (n = 3). $^{**}P < 0.01$.

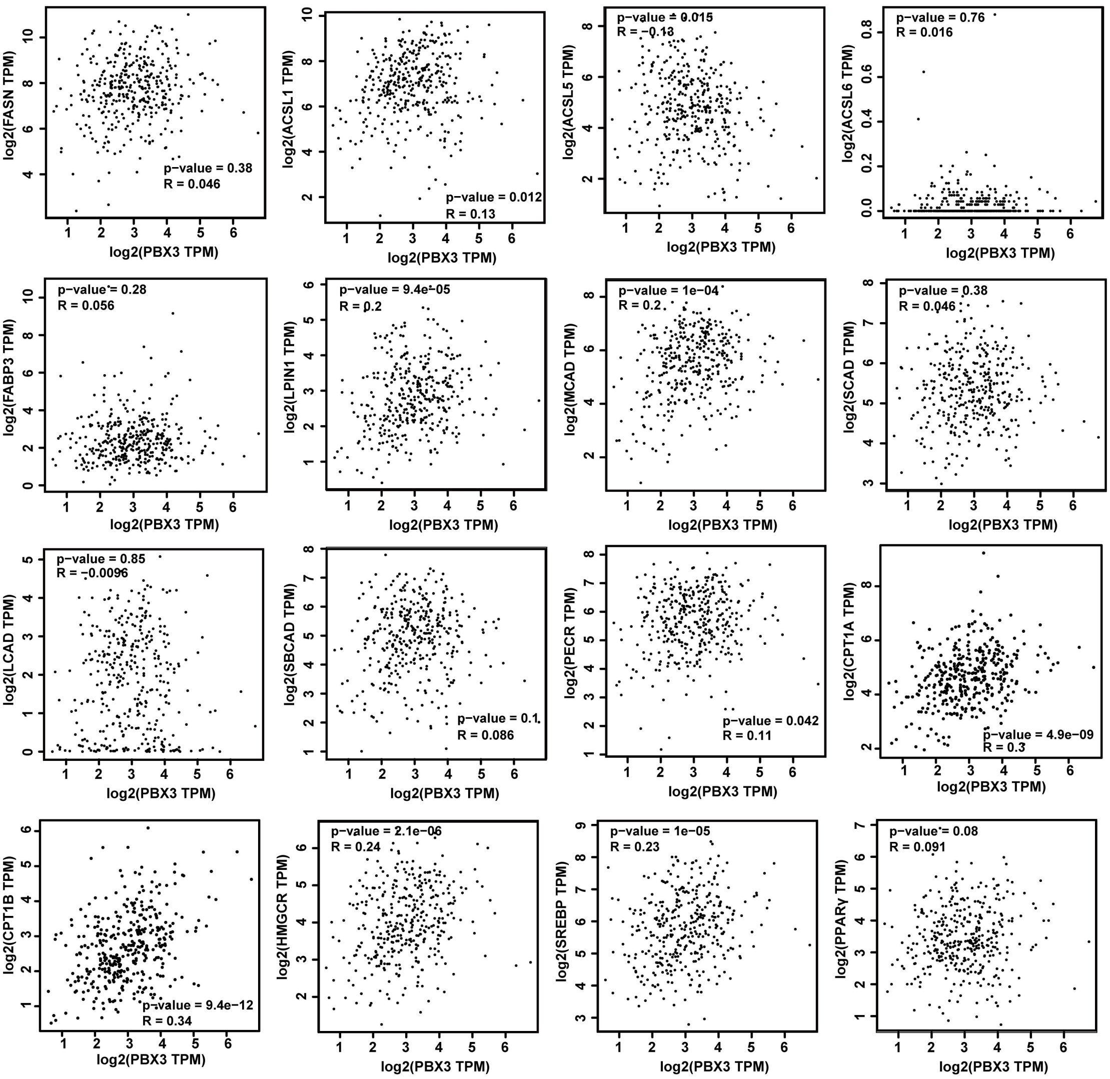

Supplemental Figure S2: Correlation of PBX3 and lipid metabolism-related genes in HCC. Correlation analysis of the expression levels of PBX3 and lipid metabolism-related genes in clinical HCC samples obtained from TCGA data set.

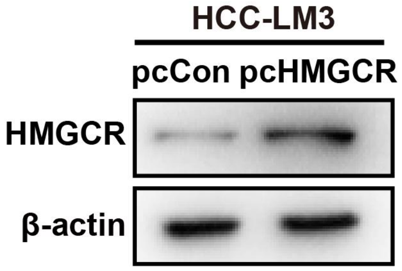

Supplemental Figure S3: Efficacy of *HMGCR* overexpression vectors.

HMGCR protein expression level in HCC-LM3 cells transfected with pcHMGCR, as examined using western blotting. Cells transfected with pcCon was used as controls. β-actin was used for western blotting loading control. pcCon: pcEF9-Puro.

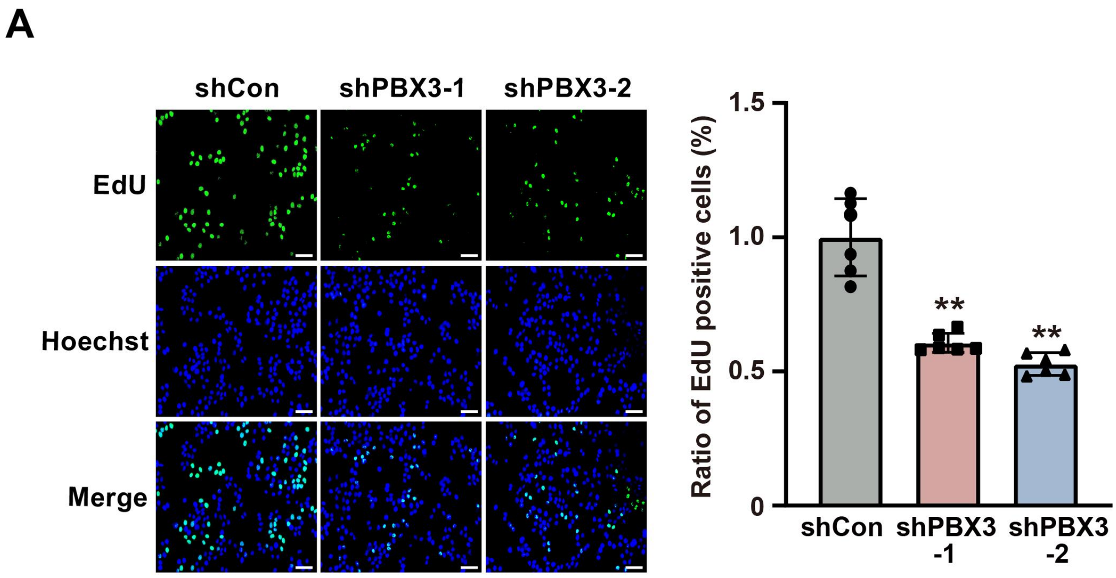

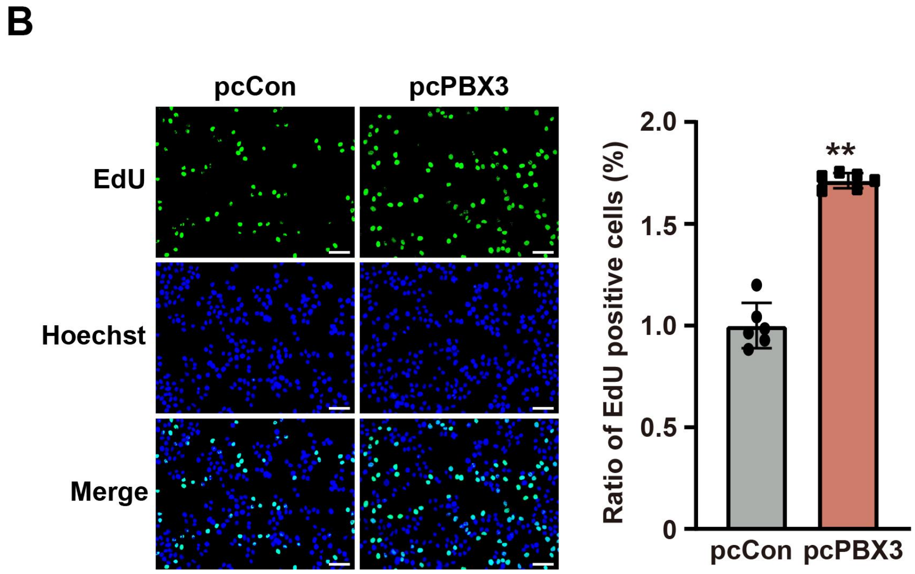

Supplemental Figure S4: PBX3 promotes HCC cells proliferation potential.

(A, B) Proliferation potential of *PBX3*-knocked down (A) and *PBX3*-overexpressing (B) MHCC-97H cells, as determined using EdU-incorporation assay. Representative images (left; scale bars: 100 μm) and quantification results (right; n = 6) are shown. Cells transfected with shCon or pcCon were used as controls. Quantification data are expressed as mean ± SD (n = 6). pcCon: pcEF9-Puro; ***P* < 0.01.

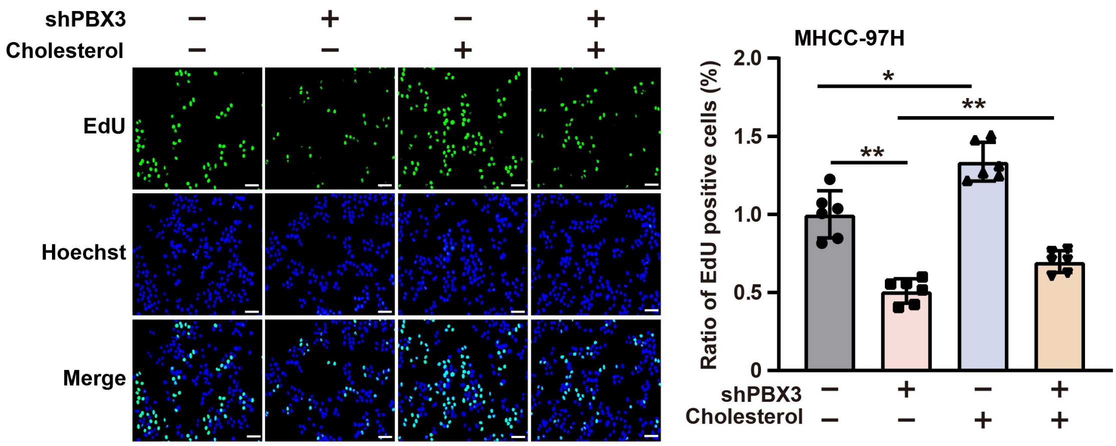

Supplemental Figure S5: Cholesterol is crucial for PBX3 regulation on HCC cells proliferation potential.

Proliferation potential of *PBX3*-knocked down MHCC-97H cells treated with cholesterol (final concentration: 10 μg/mL), as determined using EdU-incorporation assay. Representative images (left; scale bars: 100 μm) and quantification results (right; n = 6) are shown. Cells transfected with shCon was used as controls. Quantification data are expressed as mean ± SD (n = 6). ***P* < 0.01.

Supplemental Table S1. Primer pairs used for qRT-PCR.

Genes	Refseq No.	Primer sequences (5'-3')	
		Forward	Reverse
PBX3	NM_001411009.1	TACAGAAGGCCCAGGAAGTG	AAAGCTGTAGATGGTGGGCT
HMGCR	NM_000859.3	GTTCGGTGGCCTCTAGTGAG	GCATTCGAAAAAGTCTTGACAAC
FASN	NM_004104.4	AAGGACCTGTCTAGGTTTGATGC	TGGCTTCATAGGTGACTTCCA
ACSL1	NM_001995	CGACGAGCCCTTGGTGTATTT	GGTTTCCGAGAGCCTAAACAA
ACSL5	NM_203380	CTCAACCCGTCTTACCTCTTCT	GCAGCAACTTGTTAGGTCATTG
ACSL6	NM_001205251	CGCTACATCATCAATACAGCGG	GCATGGACTTAATGACCACCC
FABP3	NM_004102.4	GAGTGGGCAGAAATAACG	ATTAACAGGCTCCGAGAC
LPIN1	NM_001349208.1	TTTCCACGTCCGCTTTGGG	GTGGCCAGGTGCATAGGG
MCAD	NM_001127328	GGAAGCAGATACCCCAGGAAT	AGCTCCGTCACCAATTAAAACAT
SCAD	NM_000017	CGGCAGTTACACACCATCTAC	GCAATGGGAAACAACTCCTTCTC
LCAD	NM_001608	TGCAATAGCAATGACAGAGCC	CGCAACTACAATCACAACATCAC
SBCAD	NM_001609	GATGGCAAATGTAGACCCTACC	AAGGCCCGGAGTATCACGA
PECR	NM_018441	AACCTACCTCCCACAAAG	ATTATTCACCTCCTCCTCATT
CPT1A	NM_001876.3	ATCAATCGGACTCTGGAAACGG	TCAGGGAGTAGCGCATGGT
CPT1B	NM_152246.2	CCTGCTACATGGCAACTGCTA	AGAGGTGCCCAATGATGGGA
SREBP	NM_004176.4	CTGGTCTACCATAAGCTGCAC	GACTGGTCTTCACTCTCAATG
PPAR©	NM_138711.3	TTGCTGTCATTATTCTCAGTGGA	GAGGACTCAGGGTGGTTCAG
β-actin	NM_001101.5	CACCATTGGCAATGAGCGGTTC	AGGTCTTTGCGGATGTCCACGT

Supplemental Table S2. Antibodies used for western blotting, ChIP assay, and immunohistochemistry staining.

Antibody	Marker	Product No.	Usage	Experiment	Dilution
Anti-PBX3	Proteintech	12571-1-AP	Primary antibody	Western Blotting<br>Immunohistochemistry<br>ChIP assay<br>Immunoprecipitation	1/500<br>1/100<br>30 μg/mL cell lysate<br>25 μg/mL cell lysate
Anti-HMGCR	Proteintech	13533-1-AP	Primary antibody	Western Blotting<br>Immunohistochemistry	1/1000<br>1/200
Anti-Histone H3	Proteintech	17168-1-AP	Primary antibody	ChIP assay	30 μg/mL cell lysate
Anti-β-actin	Proteintech	60008-1-Ig	Primary antibody	Western Blotting	1/10,000
Anti-Rabbit IgG	Proteintech	B900610	Primary antibody	ChIP assay	30 μg/mL cell lysate
Goat Anti-Rabbit IgG	ZSGB-BIO	ZB2301	Secondary antibody	Western blotting	1/10,000
Goat Anti-Mouse IgG	ZSGB-BIO	ZB2305	Secondary antibody	Western blotting	1/10,000
